
JOURNAL INFORMATION
==============================
NLM Title Abbreviation: ScientificWorldJournal
Journal ID: ScientificWorldJournal
NLM Journal ID: 101131163
Journal ID: TSWJ

The Scientific World Journal

ISSN: 2356-6140
EISSN: 1537-744X
Publisher: Wiley

ARTICLE INFORMATION
==============================
PMCID: PMC5901221
PMID: 29861692
DOI: 10.1100/tsw.2007.222
Article ID: 402639
Article version: 1
Subjects: Foreword

Sponsor's Foreword

Shurtleff David 1
Ferré Sergi 2
1 Division of Basic Neuroscience and Behavioral Research, National Institute on Drug Abuse, National Institutes of Health, U.S. Department of Health and Human Services, Bethesda, MD, USA
2 Intramural Research Program, National Institute on Drug Abuse, National Institutes of Health, U.S. Department of Health and Human Services, Baltimore, MD, USA
Academic Editors: D. Shurtleff and S. Ferre

Publication date: 2007
Electronic publication date: 2007 Nov 2
Volume: 7
First page: 1
Last page: 3
Copyright: Copyright © 2007 David Shurtleff and Sergi Ferré
Copyright year: 2007
License: This is an open access article distributed under the Creative Commons Attribution License, which permits unrestricted use, distribution, and reproduction in any medium, provided the original work is properly cited.
License URL: https://creativecommons.org/licenses/by/3.0/

==============================
Foreword
NIDA Special Issue on Frontiers in Addiction Research
*TheScientificWorldJOURNAL* (2007) 7(S2), 1–3
ISSN 1537-744X; DOI 10.1100/tsw.2007.222

TheScientificWorldJOURNAL
www.thescientificworld.com

### Sponsor's Foreword

David Shurtleff[1] and Sergi Ferré[2]

*[1]Division of Basic Neuroscience and Behavioral Research, Bethesda, MD; [2]Intramural Research Program, Baltimore, MD*
*National Institute on Drug Abuse, National Institutes of Health, U.S. Department of Health and Human Services*

**E-mail:** dshurtle@mail.nih.gov; sferre@intra.nida.nih.gov

*Published November 2, 2007*

---

NIDA NATIONAL INSTITUTE ON DRUG ABUSE
National Institutes of Health
U.S. Department of Health and Human Services

Advances in neuroscience continue to fuel a revolution in our fundamental understanding of the nature of drug abuse and addiction. The growing understanding that addiction is a brain disease that is expressed as a form of compulsive behavior is influencing the direction of basic research and revitalizing drug abuse prevention and treatment efforts. The application of powerful new scientific tools and emerging technologies is accelerating advances in neuroscience research, and affording a glimpse into the mechanisms underlying the psychoactive and addicting effects of drugs of abuse. In addition, new technologies are revealing the complex interrelationship among molecular, cellular, neurobiological, and behavioral systems, and how they are altered by drugs of abuse.

National Institute on Drug Abuse (NIDA) sponsored research has supported many advances in our understanding of this disease. Indeed, NIDA's mission is to lead the Nation in bringing the power of science to bear on drug abuse and addiction. This charge has two components. The first is the strategic support and conduct of research across a broad range of disciplines. The second is ensuring the rapid and effective dissemination of the research to improve significantly the prevention, treatment, and policy related to drug abuse and addiction. The mini-review papers published in this NIDA-sponsored special issue of *TheScientificWorldJOURNAL* on "Frontiers in Addiction Research" exemplify the depth, breadth, and sophistication of the research in the field, and fulfill NIDA's mission to disseminate scientific knowledge rapidly and effectively.

The mini-reviews in this special issue are written by speakers from the 2007 NIDA-supported satellite meeting, "Frontiers in Addiction Research", held in conjunction with the annual meeting of the Society for Neuroscience (SfN), and from NIDA-supported symposia presented during the annual SfN meeting itself. The editorials in this issue, provided by symposia chairs, summarize the science and highlight research gaps and opportunities captured by the various symposia.

The four symposia from the 2007 NIDA satellite meeting include:

1. "Neuronal Adaptations and Counter-Adaptations" — which addresses the fact that the abused substances produce long-lasting changes in neuronal morphology and function. These changes are due to the pharmacological effects of drugs on the nervous system, which invoke brain systems

©2007 with author.
Published by TheScientificWorld; www.thescientificworld.com involved in reward processing and learning, among other processes. These changes may be considered neuroadaptive or neuropathological. The cellular mechanisms that regulate neural circuitry function are being uncovered and their relevance to addiction explored.

2. "Heteromerization of G-Protein-Coupled Receptors: Implications for CNS Function and Dysfunction" — focuses on the emerging area of heteromerization of neurotransmitter receptors. Several receptor classes in the CNS confer functional entities that possess different biochemical characteristics with respect to the individual components of the heteromer. Discovery of this phenomenon is changing traditional notions about neurotransmission. Receptors are now recognized to form multimolecular aggregates localized in the plasma membrane, which when stimulated, lead to functional changes in cell signaling. In this special issue, the symposium contributors review the roles of dopamine, opioid, and adenosine receptor heteromers in the CNS. Their implications for neuropsychiatric disorders, including drug addiction and agonist- and antagonist-heteromeric interactions, are discussed.
3. "Glial Cells and Addiction" — highlights recent discoveries that have led to a new appreciation of glial function. It was known previously that glia performed metabolic functions in the CNS, but recent research has shown that glia can release chemical transmitters, and play a role in synaptic formation, neural development, neuroplasticity, pain modulation, and substance abuse.
4. "Impulsivity and Addiction: A Tribute to Henri Begleiter" — co-sponsored with National Institute on Alcohol Abuse and Alcoholism (NIAAA) commemorates and complements the work of the late Dr. Henri Begleiter, who made the landmark discovery that $P_{300}$ amplitude could be used to differentiate between children of alcoholics and non-alcoholics. This result, among others, led to the exploration of alternative diagnostic phenotypes that could be linked to alcoholism susceptibility genes and their neurobiological correlates, in order to identify "endophenotypes" of the disease itself. Since endophenotypes are proximal to gene function, they provide a unique approach to the discovery of genes and genetic risk factors contributing to complex behavioral phenotypes, such as substance use disorder.

Contributions to this issue from the three NIDA-supported SfN symposia include:

1. "Understanding the Neurobiology of Drug Addiction by Studying Sleep Disturbances and Circadian Rhythms" — capitalizes on the observation that a hallmark of drug dependence, as well as other psychiatric disorders, is sleep disturbance. Drugs of abuse may alter or disrupt brain circuitry involved in regulating circadian systems, leading not only to changes in sleep, but to sleep-related cognitive function. Further, drugs of abuse can entrain circadian patterns of behavior. Thus, genes that influence circadian rhythms are implicated in the regulation of drug sensitization and reward, which may be mediated through the mesolimbic dopaminergic system.
2. "Noncoding RNAs in the Brain" — highlights the intense research efforts to identify neuronal specific RNAs and their role in post-transcriptional gene regulation during neural development and disease.
3. "Reconciling Molecular and Electrophysiological Effects of Cocaine on Neural Plasticity" — examines the physiological and molecular changes in the nucleus accumbens that show evidence of hypoactivity following cocaine exposure and other changes indicative of enhanced responsiveness, such as changes in glutamate receptor distribution, signaling, and gene expression. This paradox is discussed in this issue.

In summary, while not exhaustive of the neuroscience research that NIDA supports, this special issue highlights the innovation, breadth, and diversity of the NIDA neuroscience research portfolio. It also exemplifies the need for NIDA to continue to support a variety of basic and clinical neurobiological investigations in order to uncover the ever-elusive mechanisms that contribute to drug addiction. At the same time, the research that NIDA supports must be applied to and translated into advances in and the development of new medications and therapies that will have a direct impact on this serious public health problem. Investment in basic and clinical research such as that highlighted in this special issue will contribute to this effort.

We wish to thank the Managing Editor for TSWJ, Tara Packer, for her invaluable assistance throughout the review and editorial process, and the Publisher of TSWJ, Graham V. Lees, for his support and insight in developing this special issue. We particularly thank those symposia presenters and symposia chairs who took the time to write and contribute to this issue. Finally, a special thanks to Rita Liu and Cathrine Sasek, co-Chairs of NIDA's Neuroscience Consortium, for guiding and coordinating the overall content of NIDA's neuroscience program at the SfN meeting.

For further information on NIDA's mission and research funding opportunities, visit NIDA's website at www.nida.nih.gov. For information about NIAAA please visit www.niaaa.nih.gov.

**This article should be cited as follows:**

Shurtleff, D. and Ferré, S. (2007) Sponsor's foreword. *TheScientificWorldJOURNAL* **7(S2)**, 1–3. DOI 10.1100/tsw.2007.222.
